# Metformin may improve the outcome of patients with colorectal cancer and type 2 diabetes mellitus partly through effects on neutrophil extracellular traps

**DOI:** 10.1038/s44276-023-00022-w

**Published:** 2023-12-12

**Authors:** Akira Saito, Koji Koinuma, Rie Kawashima, Hideyo Miyato, Hideyuki Ohzawa, Hisanaga Horie, Hironori Yamaguchi, Hiroshi Kawahira, Toshiki Mimura, Joji Kitayama, Naohiro Sata

**Affiliations:** 1 Department of Gastrointestinal Surgery, Jichi Medical University, Shimotsuke, Japan; 2 Department of Oral and Maxillofacial Surgery, Jichi Medical University, Shimotsuke, Japan; 3 Department of Clinical Oncology, Jichi Medical University, Shimotsuke, Japan

## Abstract

**Background:**

Although metformin reduces the risk of cancer-related mortality in patents with type 2 diabetes, the mechanism of its anti-cancer effects has not been fully understood.

**Method:**

Impact of metformin on survival was examined in patients who underwent curative colectomy for colorectal cancer (CRC). The effects of metformin in neutrophil extracellular traps (NETs) were examined with in-vitro experiments and multiplex immunohistochemistry of surgically resected CRC specimens.

**Results:**

Prior intake of metformin prolonged relapse-free (*P* = 0.036) and overall survival (*P* = 0.041) in 289 patients with T2DM to the comparable levels to those of 1576 non-diabetic patients. Metformin reduced the production of NETs stimulated with lipopolysaccharide or HT-29 colon cancer cells to 60% of control. Neutrophils markedly suppressed the chemotactic migration of activated T cells in an NET-dependent manner, which was reversed by metformin treatment up to approximately half of the migration without neutrophils. Immunohistochemical analysis revealed a significant association between metformin intake and a reduction in the numbers of tumor-associated neutrophils (TANs) and NETs. Simultaneously, metformin intake was found to increase the presence of CD3(+) and CD8(+) tumor-infiltrating T cells (TILs), particularly at the tumor-invasion front, especially in areas with fewer TANs and NETs.

**Conclusion:**

Metformin suppresses the diabetes-associated enhancement of NET formation, which can augment the infiltration of TILs in CRC tissues. The anti-tumor effect of metformin in patients with T2DM may be, at least partly, attributable to the inhibition of NETs.

## Introduction

Type 2 diabetes mellitus (T2DM) is the most common chronic and metabolic disease, and growing evidence supports a biologic or physiologic link between T2DM and cancer [1]. Many epidemiologic studies have demonstrated that T2DM increases the risk of development and mortality for various cancers, including colorectal cancer (CRC) [2–4]. Key contributors to tumorigenesis and tumor progression include hyperglycemia, insulin resistance, and increased levels of insulin and insulin-like growth factors as well as increased adipocytokines associated with obesity [2, 3, 5].

Recent studies have also suggested that anti-diabetic agents can greatly influence tumor behavior in diabetic patients with cancer [6, 7]. In particular, metformin is well known to reduce the incidence of cancer development and cancer-related mortality, although the degree of reduction varies among types of cancer [8–11]. In preclinical studies, metformin directly suppressed tumor cell survival and proliferation through 5-AMP-activated protein kinase (AMPK)-dependent and -independent mechanisms [12–14]. More recent studies suggest that the anti-tumor effects of metformin are largely dependent on the enhancement of host immune responses against cancer [15, 16]. In murine studies, metformin upregulated T cell-mediated immune responses [17–19] and mediated the repolarization of macrophages from the M2 to the M1 phenotype in the tumor microenvironment [18, 20, 21], thus potentially inhibiting tumor growth. Furthermore, metformin has been shown to downregulate programmed cell death receptor ligand 1 in tumor cells, thereby enhancing T-cell mediated cytotoxicity [22–24]. However, the mechanisms underlying how metformin modulates the host immune system remain to be elucidated.

Neutrophil extracellular traps (NETs) are complex structures composed of unfolded DNA that is decorated with histones, proteases, and granular and cytosolic proteins. NETs are recognized as an important antimicrobial mechanism to immobilize and kill pathogens [25]. Recent studies suggest that NETs promote tumor progression and metastasis by capturing circulating tumor cells [26–28], inducing epithelial–mesenchymal transition (EMT) [29], awakening dormant cancer cells [30], and stimulating cancer-associated thrombosis [31]. In addition, NET markers are markedly elevated in the circulating blood of patients with T2DM [32–35]. This finding suggests that neutrophils in diabetic patients are more likely to produce NETs than those in non-diabetic people, and this difference in NET production might be mechanically associated with the incidence and progression of cancer. Therefore, in the current study, we examined the effects of metformin on NET formation in human samples.

## Materials and methods

### Monoclonal antibodies (mAbs), reagents and cell line

MAbs to CD3 (dilution, 1:150) and CD66b (dilution, 1:600) were purchased from Thermo Fisher Scientific (Waltham, MA), mAb to CD8α (dilution, 1:2000) from Proteintech Group (Rosemont, IL), and mAb to histone H3 (Cit-H3, dilution, 1:500) from Abcam (Cambridge, United Kingdom). Signal enhancer HIKARI for Immunostain Solution B, antibody dilution buffer, and blocking solution One-Histo (catalog no. 06349-64) were purchased from Nacalai Tesque (Kyoto, Japan). Lipopolysaccharide (LPS) and metformin hydrochloride were purchased from Sigma–Aldrich (St. Louis, MO) and Wako Chemical (Tokyo, Japan), respectively. To bind extracellular DNA components, SYTOX Green was purchased from Thermo Fisher Scientific. Detailed were expressed in supplementary Table 2.

The human colon cancer cell line, HT-29, was obtained from the RIKEN cell bank (Tsukuba, Japan). The cells were expanded by the culture with Dulbecco’s Modifed Eagle Medium (DMEM) supplemented with 10% fetal bovine serum (FBS), 100 units/ml penicillin and 100 mg/ml streptomycin as well as 5 mg/ml Plasmocin (INVIVOGEN, SanDiego, CA) to prevent mycoplasma infection. Subsequently, these cells were cryopreserved in liquid nitrogen. Upon thawing, we verified the absence of mycoplasma contamination in the newly thawed cells. For our experiments, we exclusively employed cells that had undergone fewer than two passages to minimize phenotypic variation. While we did not perform additional genomic authentication, we assessed the identity of the thawed cells through visual inspection and growth curve analysis. and stored in liquid nitrogen. To limit phenotypic drift, cells passaged fewer than three times were used for the experiment.

### Patients and samples

From April 2010 through March 2021, 1865 patients with Stage I to III colorectal cancer (CRC) underwent radical colectomy with curative intent in the Department of Surgery, Jichi Medical University Hospital (Tochigi, Japan). Of these, 289 patients (15.5%) had T2DM at the time of surgery, and 62 of these 289 patients (21.5%) were receiving metformin. The remaining 1576 patients with CRC comprised the T2DM-free group. Data for gender, age, medical history, treatment of diabetes, surgical procedure, preoperative laboratory test results, pathologic evaluation (histologic tumor type, depth of tumor, nodal metastasis, vascular invasion, and lymphatic invasion) and postoperative outcomes were extracted from the electronic medical record after the patient provided written informed consent. This study was approved by the Institutional Review Board of Jichi University Hospital (approval no., clinic A21-064) and was conducted in accordance with the guiding principles of the Declaration of Helsinki.

Among the 62 surgically resected CRC tumors from patients treated with metformin, the most recent 40 specimens were used for immunostaining. In addition, samples from 40 patients who did not receive metformin were selected from among 221 cases through propensity score matching and used as a control group for immunostaining. Gender, age, tumor site, histologic type, pT category, pN category, pStage, lymphatic invasion, venous invasion, and use of adjuvant therapy were selected for score matching.

### NET formation assay

NET formation was evaluated by using neutrophils purified from the peripheral blood of healthy donors, as described previously [36]. In brief, after dextran sedimentation, leukocyte-enriched plasma was overlaid on Ficoll–Hypaque solution (Cytiva, Uppsala, Sweden) and centrifuged at 800 g for 15 min. The bottom layers were collected and washed twice with PBS containing 0.02% EDTA. Red blood cells were removed with RBC Lysis Buffer (BioLegend, San Diego, CA), and remaining cells were washed twice with PBS containing 0.02% EDTA. The polymorphonuclear leukocyte fractions, which comprised ≥95% neutrophils, were used for following experiments. Neutrophils were adjusted to 1 × 10^6^ cells per well by using serum-free RPMI1640 medium, seeded into 6-well plates (Thermo Scientific, Seoul, Korea), and incubated with LPS (final concentration, 5 µg/ml) at 37 °C for 2 h; metformin (maximum final concentration, 100 µM) was added to some wells. To assess NET production, SYTOX Green nucleic acid stain (final concentration, 50 nM; Thermo Fisher Scientific) was added to wells, and plates were read by using a fluorescence stereomicroscope (model BZ8000, Keyence, Osaka, Japan). To quantify NET, cells that showed the characteristic SYTOX Green strand staining were counted, and the ratio of NET cells to total neutrophil count was calculated for 3 randomly selected fields.

### Migration assay

Chemotactic migration of activated T cells was evaluated by using a two-chamber system with 3-µm–pore Transwell inserts (Corning, Corning, NY). Peripheral blood mononuclear cells from healthy volunteers were cultured for 5 to 7 d in RPMI 1640 containing recombinant interleukin 2 (final concentration, 10 ng/l; Thermo Fisher Scientific) and 10% fetal bovine serum on plates coated with anti-CD3 mAb. Activated T cells were labeled with calcein-AM (Thermo Fisher Scientific) and resuspended in DMEM containing 0.1% bovine serum albumin. HT-29 human colorectal carcinoma cells (1 × 10^4^ cells per well) were cultured in a 24-well culture plate containing DMEM supplemented with 10% fetal calf serum. After cells became confluent, the medium in each well was replaced with 500 μl DMEM supplemented with 0.1% bovine serum albumin. Activated T cells (1 × 10^5^ cells per well) were seeded into the culture inserts, which were placed in the lower chamber containing the HT-29 cells. In some wells, neutrophils were preincubated with DNAse I (100 U/ml) or metformin (100 μM) for 30 min and added to culture inserts containing activated T cells.

After 2 h, the cells that had transmigrated to the lower chamber were recovered and counted by using flow cytometry, as described previously [37]. In brief, the culture inset was removed, and the cells that had migrated into the lower chamber were collected in a FACS tube, washed with 2 ml PBS, resuspended in 300 μl PBS, acquired for 1 min during a 60 μl/min run of flow cytometry, and counted as the number of calcein-positive T cells. As a 10% control, 1 × 10^4^ cells were added to the lower chamber and counted in the same way, and percentage migration was calculated against the 10% control value.

### Multiplex immunohistochemistry and image processing and analysis

All specimens were fixed in formalin, embedded in paraffin, cut into sections (thickness, 4 µm), and then used for multiplex immunohistochemistry. The staining protocol has been described previously [38]. In brief, after deparaffinization, the sections were stained with hematoxylin for 1 min, followed by whole-tissue scanning (OlyVIA SlideView VS200, Olympus, Tokyo, Japan). After imaging, endogenous peroxidases were blocked in 0.3% hydrogen peroxide for 30 min. For antigen retrieval, the sections were processed by microwaving in 10 mM sodium citrate buffer (pH 6.0) for 10 min. After incubation with a blocking agent (Blocking One Histo) for 10 min at room temperature to prevent nonspecific binding, the sections were incubated with primary antibodies for 30 min at room temperature, thoroughly washed with Tris-buffered saline containing 0.1% Tween 20, and stained (Histofine Simple Stain PO(M) Kit for Rabbit or Mouse, Nichirei, Tokyo, Japan). Primary antibody binding was visualized by using an ImmPACT AMEC Red Substrate Kit (Vector Laboratories, Newark, CA), and sections were scanned (OlyVIA SlideView VS200). Stained slides were then dehydrated through an alcohol gradient (90% ethanol, 80% ethanol, and 70% ethanol; 2 min at each concentration) until no visible 3-amino-9-ethylcarbazole reaction product remained. After rehydration, antibodies were eluted by incubating sections in 10 mM sodium citrate buffer (pH 6.0) for 10 min by using the microwave method. Complete stripping of antibodies and signals was confirmed throughout all cycles (Supplementary Fig. 1). The sections were then stained from the blocking step with another antibodies. To validate the specificity of the antibody and confirm signal removal between sequential immunohistochemical staining steps, we employed two types of negative controls, i.e, conventional negative control slides treated with 2.5% goat serum in PBS without the addition of primary antibodies and additional sequential IHC negative controls.

After chromogenic sequential immunohistochemical staining, each image was converted to digitized pseudo-colored image. Those images were sequentially co-registered to ensure precise alignment of cell features at the single-pixel level, utilizing the CellProfiler version 2.1.1 pipeline ‘Alignment Batch.cppipe,’ available under General Public License version 2.0 according to the manufacturer’s recommendation. Finally, all the images were merged using ImageJ Fiji (National Institutes of Health, Bethesda, MD) (Supplementary Fig. 2). To assess cell density cells, we counted the cells present in 3 randomly selected regions of interest (0.5 × 0.5 mm^2^) of tumor stroma. Analysis was blinded with respect to clinical outcomes by 3 investigators.

### Statistical analysis

Statistical analyses were performed by using Prism 9 (GraphPad Software, San Diego, CA). Statistical differences in clinical and pathologic factors between groups were evaluated by using the Mann–Whitney *U* test or Fisher’s exact test. Survival was calculated according to the Kaplan–Meier method, and differences were evaluated by using the log-rank test. In all analyses, the standard for significant difference was set as *P* < 0.05.

## Results

### The outcome of CRC patients with T2DM differs depending on their metformin treatment

**Fig. 1:**
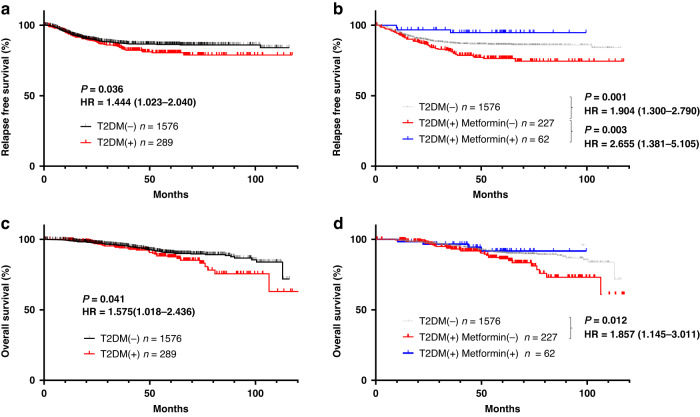
Metformin intake improves prognostic outcomes in colorectal cancer (CRC) patients with type 2 diabetes mellitus (T2DM) who underwent curative surgery. **a** Relapse-free survival (RFS) and (**b**) overall survival (OS) after surgery for colorectal cancer in patients with (*n* = 1576) or without (*n* = 289) T2DM were evaluated according to the Kaplan–Meier method and *P* values were calculated by using the log-rank test. **c** RFS and (**d**) OS of the patients with T2DM who were taking (*n* = 62) or not taking (*n* = 227) metformin at colectomy. Gray lines show the survival curves of the patients without T2DM.

We first examined the impact of metformin intake on the cancer-associated outcomes of patients with T2DM who underwent curative surgery for CRC. Relapse-free survival (RFS; Fig. 1a) and overall survival (OS; Fig. 1c) were significantly shorter in the 289 patients who had T2DM at surgery than in the 1576 patients who did not (RFS: HR = 1.444, 1.023–2.040, *P* = 0.036, OS:HR = 1.576, 1.018–2.436, *P* = 0.041). However, the CRC-associated outcomes of the diabetic patients differed markedly depending on whether they received metformin. Among the 289 patients with CRC and T2DM, 62 were being treated with metformin at the time of surgery. These 62 patients were younger and had higher HbA1c levels than the 227 diabetic patients without metformin treatment (Table 1), but none of the other factors differed significantly between these groups. Both RFS and OS after CRC were significantly shorter in the 227 diabetic patients without metformin treatment than in the 1576 without T2DM (RFS: HR = 1.904, 1.300–2.790, *P* = 0.001, OS: HR = 1.857, 1.145–3.011, *P* = 0.012). In contrast, RFS after surgery for CRC was significantly longer for the metformin-treated diabetic patients than for those without metformin (HR = 2.655, 1.381–5.105, *P* = 0.003) and even tended to be better as compared with those without T2DM although not significantly (*P* = 0.203) (Fig. 1b). Furthermore, the OS curve for metformin-treated patients with T2DM who underwent surgery for CRC was nearly parallel to that of their nondiabetic peers (Fig. 1d). Multivariate analysis with cox regression method showed that metformin use was an dependent prognostic factor in the whole patients (Supplementary Table 2).

**Table 1:** Clinical and pathologic features of CRC patients with T2DM.

	Metformin(−) *n* = 227	Metformin(+) *n* = 62	*P*
Gender (female/male)	150/77	43/19	0.63
Age (y)	70 (42–95)	67 (42–83)	<0.01
HbA1c (%)	6.7 (5.1–9.9)	6.9 (5.3–11.3)	0.023
Tumor site (right/left)	79/148	18/44	0.45
Pathology (well/poor)	213/14	60/2	0.54
pT category (1/2/3/4)	53/32/79/63	16/12/26/8	0.08
pN category (−/+)	8/219	5/57	0.16
Lymphatic invasion (−/+)	126/101	36/26	0.72
Venous invasion (−/+)	73/154	21/41	0.80
pStage (I/II/III)	75/77/75	24/24/16	0.27
Neoadjuvant therapy (−/+)	8/219	5/57	0.16
Adjuvant therapy (−/+)	42/185	13/49	0.72

### Metformin inhibits the production of NETs induced by LPS

**Fig. 2:**
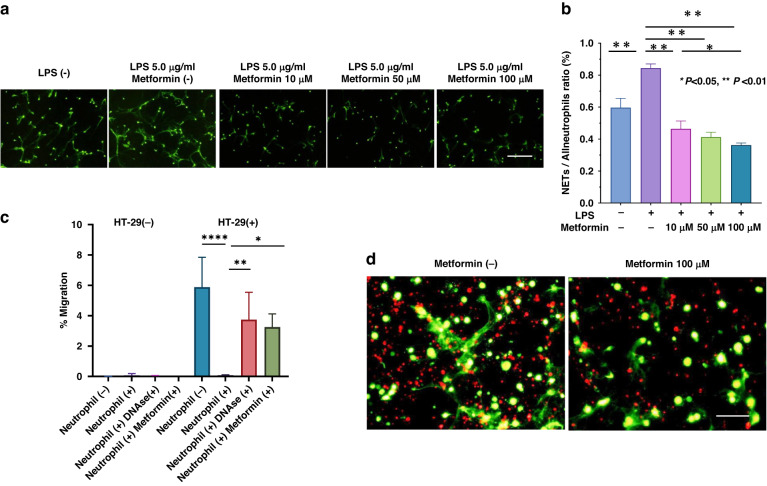
Metformin alleviates impaired lymphocyte migration impairment by suppressing neutrophil extracellular trap (NET) formation. **a** Neutrophils were purified from the peripheral blood of healthy volunteers and cultured in RPMI supplemented with LPS with or without metformin (10 to 100 μM). After 3 h, SYTOX Green was added, and the plate was viewed under a fluorescence stereomicroscope. Scale bars, 100 μm. **b** Neutrophils that showed characteristic strand staining for SYTOX Green were counted as NETs, and their ratios relative to total neutrophil counts were calculated in 3 randomly selected fields. Data are shown as the mean ± 1 SD of triplicates from one of two experiments. *P < 0.05, **P < 0.01. **c** Peripheral blood mononuclear cells, which consisted primarily of activated T cells, were cultured for 5 to 7 d in the presence of 10 ng/ml recombinant interleukin 2 on anti-CD3 mAb-coated plates. Cells were stained with calcein-AM, and 1 × 10^5^ stained cells were seeded into an upper chamber (3-μm pores); the cell-containing upper chambers were placed on the lower chambers, which contained or did not contain HT-29 cells. After 2 h, the proportion of T cells that migrated to the lower chamber was quantified by using flow cytometry as described in Materials and Methods. In some wells, neutrophils were preincubated with DNAse I (100 U/ml) or metformin (100 μM) for 30 min and added to the culture inserts containing activated T cells. Data are shown as mean ± 1 SD of triplicates from one of 3 experiments. *P < 0.05, **P < 0.01, ***P < 0.001 (**d**) Activated T cells were stained with PKH26, and the migration assay was performed in the presence of neutrophils with (lower panel) or without (upper panel) 100 mM metformin. After 2 h, the membranes of the culture inserts were stained with SYTOX Green. Photos taken under the wavelength for FITC (SYTOX Green) and rhodamine (PKH26) were superimposed. Scale bar, 50 μm.

Fluorescence microscopy after SYTOX green staining revealed many thread-like NET structures among human neutrophils that had been stimulated with LPS in vitro. The addition of metformin (10 to 100 μM) to the neutrophil cultures reduced NET formation in a dose-dependent manner up to 60% of control (Fig. 2a, b).

### Metformin restores the neutrophil-inhibited chemotactic migration of activated T cells to HT-29 cells

**Fig. 3:**
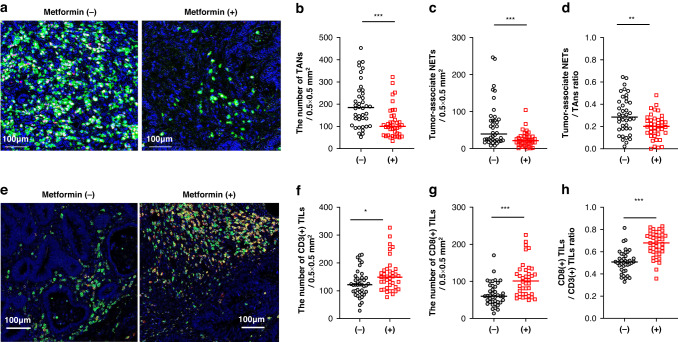
Metformin attenuates the density of tumor-associated neutrophil extracellular traps (NETs) while enhances the density of tumor-infiltrating lymphocytes (TILs) in the colorectal cancer (CRC) tissue. **a** Representative image of multiplex immunostaining of tumor-associated neutrophils (TANs) and neutrophil extracellular traps (NETs) at the invasive front of colorectal cancer. Images stained for hematoxylin (blue), CD66b (green), and Cit-H3 (red) were merged. Arrowheads show CD66b(+) Cit-H3(+) NETs. The densities of TANs (**b**) and NETs (**c**), and the NET:TAN ratio (**d**), were calculated as described in the Materials and Methods and compared between 40 propensity-score–matched tumors from each group. **e** Representative image of multiplex immunostaining of tumor-infiltrating T cells (TILs). Images stained for hematoxylin (blue), CD3 (green), and CD8 (red) were merged. Arrows point to CD3(+)CD8(–) TILs, and arrowheads indicate CD3(+)CD8(+) TILs. The densities of CD3(+) TILs (**f**) and CD3(+)CD8(+) TILs (**g**), and the CD8(+):CD3(+) TIL ratio (**h**) were calculated. *P* values were calculated by using the Mann–Whitney *U* test. *P < 0.05, ***P < 0.001.

Although they showed little spontaneous migration, activated T cells robustly migrated to HT-29 cells cultured in the lower chamber of the two-chamber system (Fig. 3a). However, adding neutrophils to the upper chamber completely inhibited the chemotactic migration of the T cells to the lower chamber containing HT-29 cells. Migration was recovered by adding DNAse I to the neutrophil–T cell chamber to degrade NETs. Similarly, preincubation of neutrophils in 100 μM metformin restored T cell migration to the same level as seen after DNAse I treatment (Fig. 2c). Labeling T cells with PKH26 and staining the membranes of culture inserts with SYTOX Green at the end of the migration assay revealed that many T cells remained in close contact with NETs that formed in the upper chamber (Fig. 2d, left panel). This finding indicates that HT-29 cells stimulate the production of NETs, which physically trap the T cells and thus inhibit their chemotactic migration. However, preincubating neutrophils in 100 μM metformin markedly decreased the number of NETs present (Fig. 2d, right panel), suggesting that metformin restores T cell migration by inhibiting NET formation.

### Metformin intake decreases the number of tumor-associated neutrophils (TANs) and NETs in CRC tissues from patients with T2DM

To examine whether metformin intake alters the tumor immune microenvironment, we used multicolor immunohistochemistry to evaluate the density of neutrophils and NET formation at the invasive tumor front of surgically resected CRC tissue. Using propensity score matching among the diabetic patients with CRC who had been treated with or without metformin, we selected 40 patients from each group who underwent surgery during the same period (Table 2). TANs were defined as green-stained cells that expressed CD66b mainly at the cell membrane (Fig. 3a). Among TANs, cells that showed red staining for Cit-H3 in the nuclear area were defined as NETs. The density of TANs was significantly lower in metformin(+) tumor tissue (Median, 100.0 TANs/0.5 × 0.5 mm^2^; range, 34.0–322.3) than in metformin(−) samples (Median, 169.7 TANs/0.5 × 0.5 mm^2^; range, 36.0–340.3; *P* < 0.001) (Fig. 3a, b). Similarly, the density of NETs was lower in metformin(+) tumors (Median, 12.3 cells/ 0.5 × 0.5 mm^2^; range, 0–42.7) than in metformin(−) samples (Median, 27.3; range, 7.3–76.0; *P* < 0.001), with a lower proportion of NETs among total TANs (metformin(+): median, 18.7%; range, 2.0–34.3%; metformin(−): median, 20.3%; range, 0–26.7%; *P* < 0.01) (Fig. 3a, c, d).

**Table 2:** Clinical and pathologic features of CRC patients with T2DM examined with immunohistochemistry.

	Metformin(–) *n* = 40	Metformin(+) *n* = 40	*P*
Tumor site (right/left)	18/32	16/34	0.82
Pathology (well/poor)	39/1	39/1	>0.99
pT category (1/2/3/4)	10/5/14/11	7/8/18/7	0.80
pN category (−/+)	32/8	32/8	>0.99
pStage (I/II/III)	14/18/8	13/19/8	>0.99
Lymphatic invasion (−/+)	27/23	28/22	>0.99
Venous invasion (−/+)	11/29	10/30	>0.99
Adjuvant therapy (−/+)	35/5	33/7	0.76

### Metformin intake increases the number of tumor infiltrating T cells (TILs) in CRC with T2DM

We immunostained the same specimens as for the TAN analysis with mAbs to CD3 and CD8 to examine tumor-infiltrating lymphocytes (TILs). CD3(+) T cells were visualized as green signals, whereas yellow signals in the dual-stain imaging represented CD8(+)CD3(+) cells (Fig. 3e). The density of CD3(+) TILs was significantly higher in metformin(+) samples (Median, 148.7 cells/0.5 × 0.5 mm^2^; range, 77.2–326.8) than in metformin(–) tumors (Median, 121.9 cells/0.5 × 0.5 mm^2^; range, 28.6–229.8; *P* < 0.05) (Fig. 3e, f). Likewise, the number of CD8(+) TILs was increased more prominently in metformin(+) tumors (Median, 101.1 cells/0.5 × 0.5 mm^2^; range, 49.4–225.2) than in metformin(–) tissue (Median, 59.9 cells/0.5 × 0.5 mm^2^; range, 13.6–170.4; *P* < 0.001) (Fig. 3g). Finally, the ratio of CD8(+):CD3(+) TILs was significantly higher in metformin(+) tumors (Median, 67.9%; range, 35.8–82.9%) than in metformin(–) specimens (Median, 50.7%; range, 32.9–81.4%; *P* < 0.001) (Fig. 3h).

### The distribution pattern of TILs is opposite to those of TANs and NETs

**Fig. 4:**
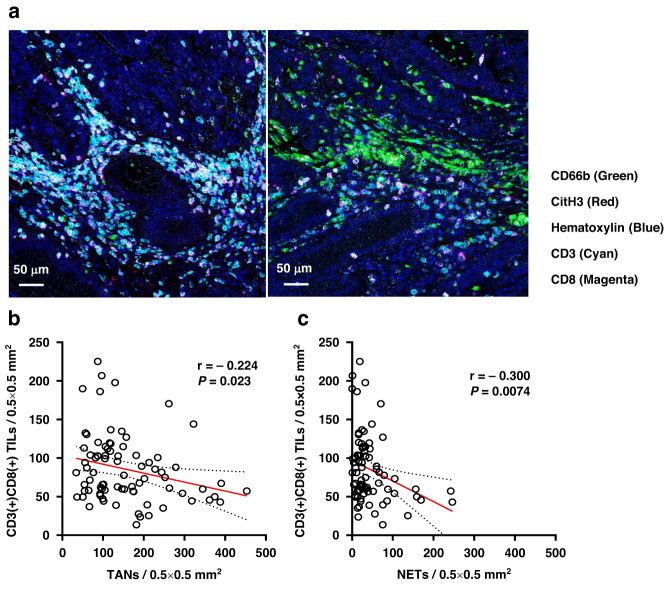
An inverse correlation between neutrophil extracellular traps (NETs) infiltration and CD8-(+) T lymphocytes infiltration in colorectal cancer (CRC) tissues. **a** Images of multiplex immunostaining of TANs, NETs, and TILs in different regions of the colorectal tumor specimen from a representative patient. Five images stained for hematoxylin (blue), CD66b (green), Cit-H3 (red), CD3 (cyan), and CD8 (magenta) were merged. Correlation between the density of CD3(+)CD8(+) TILs and that of TANs (**b**) or NETs (**c**) among all 80 tumor samples evaluated. Pearson’s simple linear regression analysis was used to calculate *r* and *P* values.

Using a multiple image processing strategy, we next examined the spatial distribution of TILs in the same CRC tumor specimens as evaluated earlier. In most tumor samples, CD3(+)CD8(+) TILs were mainly detected in areas where TANs and NETs were sparse, whereas relatively few TILs infiltrated in areas with abundant TANs and NETs (Fig. 4a). When we examined the correlation of the densities of TANs, NETs, and TILs in all 80 tumor samples from T2DM(+) patients treated for CRC, the density of CD8(+) TILs showed weak inverse correlation with that of TANs (*r* = −0.224, *P* = 0.023) (Fig. 4b). Similarly, the density of CD8(+) TILs was inversely related to that of NETs, but was somewhat stronger than for TANs (*r* = −0.300, *P* = 0.0074) (Fig. 4c).

## Discussion

In addition to its anti-diabetic effects, metformin has various pharmacologic properties, including anti-oxidative, anti-inflammatory, and anti-cancer effects [39]. In particular, the results of numerous epidemiologic studies support metformin’s anti-tumor effect [8–11]. In the current study, we confirmed that concomitant diabetes worsened the RFS and OS of patients who received curative surgery for CRC. However, metformin intake before surgery remarkably improved the CRC-associated outcomes of diabetic patients to a level equal to (OS) or even better (RFS) than that of non-diabetic patients. Unraveling the anti-tumor effect of metformin has been approached from many directions. Recent experimental reports suggest that the anti-tumor properties of metformin are largely dependent on the enhancement of host immune responses [15–18, 20–24]. However, the mechanisms underlying these immunomodulatory effects are not fully elucidated.

Neutrophils are the most abundant cell type among circulating leukocytes and they play crucial roles not only in the innate immune system but also in the adaptive immune response against cancer [40, 41]. Neutrophil responses to infection—including chemotaxis, phagocytosis, and the intracellular production of reactive oxygen species (ROS) production—are dysregulated in patients with T2DM, thus resulting in increased chronic and recurrent infections [42, 43]. In contrast, the generation of NETs and pro-inflammatory cytokines are upregulated in diabetic patients, causing damage by perpetuating inflammation [32–35]. A recent study demonstrated that metformin reduces the generation of ROS via the PKC–NADPH oxidase pathway, thereby inhibiting PMA-induced formation of NETs [44]. In the current study, we demonstrated that metformin suppresses the production of NETs induced by LPS or through co-culture with HT-29 cells in vitro. These results suggest that metformin reduces ROS production by various stimuli, thus inhibiting NET formation.

In migration experiments, we found that the co-culture of neutrophils with HT-29 colon cancer cells in a double-chamber system induced abundant NET formation; these NETs efficiently trapped activated T cells and strongly inhibited their chemotactic migration. We recently reported that activated neutrophils strongly inhibit the chemotaxis of activated T cells to CXCL11 through various mechanisms, including H_2_O_2_ generation, CXCL11 degradation, and physical trapping [45]. The results of the current study are consistent with our previous study and strongly suggest that tumor cells induce NET production, which suppresses the infiltration of effector T cells into tumor tissue. This suppression of T cell influx might be an important component of tumor immune escape and an additional means by which NETs promote tumor progression. In fact, Teijeira et al. have recently demonstrated that NETs can wrap tumor cells and shield them from cytotoxicity by obstructing their contact with cytotoxic immune cells [46].

More importantly, the addition of metformin largely restored the chemotactic migration of activated T cells and reduced NET formation. Immunohistochemical results clearly showed that the densities of TANs and NETs were reduced in the CRC tissues of diabetic patients who were treated with metformin. This finding agrees with the results of other recent studies indicating that the presence of NETs in the tumor site correlates with worse prognosis in various types of cancers including gastric [47], pancreatic [48], and cervical [49] cancers. In contrast and as described in our previous study [50], the densities of CD3(+) and CD8(+) TILs were significantly increased in diabetic CRC patients treated with metformin. In the current study, we also found that TILs were particularly prominent in areas where NETs were rare and that the density of TILs was negatively correlated with those of TANs and NETs in the entire subset of patients evaluated. The immunohistochemistry data are completely consistent with the results of the in vitro experiments and strongly suggest that metformin might increase TILs by reducing NET formation in CRC tissue.

This study is constrained by certain limitations as it is a single-center, retrospective study with a limited number of patients. However, when interpreted in conjunction with the in vitro experimental findings, it suggests that metformin may have the potential to mitigate the accumulation of TANs and NETs in diabetic patients. This, in turn, could bolster the infiltration of effector T cells and bring about a significant transformation in the tumor microenvironment, shifting it from an immunosuppressive state to an immunocompetent one. Recent clinical studies have suggested the efficacy of metformin as an anticancer agent, especially when provided in combination with immune checkpoint inhibitors (ICIs) [51, 52]. These findings seem reasonable, because the clinical efficacy of ICIs is highly dependent on TIL density [53, 54]. In another study, metformin suppressed NET formation and inhibited the development of pancreatic cancer in obese mice [55]. Together, these results suggest to us that the anti-tumor effect of metformin is dependent, at least partly, on the inhibition of NETs. In fact, metformin is attracting much attention as a candidate for drug reposition for NET-related vascular [56, 57] and neurodegenerative [58, 59] diseases.

## Supplementary information

Supplementary Information
