# Supplementary material for: Metformin may improve the outcome of patients with colorectal cancer and type 2 diabetes mellitus partly through effects on neutrophil extracellular traps
Source: BJC Rep. 2023 Dec 12;1:20. doi: 10.1038/s44276-023-00022-w (PMC11524073; doi:10.1038/s44276-023-00022-w)
Supplement: Supplementary file 1 — Supplementary Information [file 44276_2023_22_MOESM1_ESM.docx]

**Supplementary Table1. Materials**

| Reagents and antibodies | Clone | Manufacturer | Catalog numbers |
| --- | --- | --- | --- |
| Lipopolysaccharide (LPS) | N/A | Sigma–Aldrich | L2630-10MG |
| Metformin | N/A | Wako Chemical | 136-18662 |
| Recombinant interleukin 2 | N/A | Thermo Fisher Scientific | AF-200-02 |
| Calcein-AM sulution | N/A | Dojindo | C396 |
| PKH26 | N/A | Sigma-Aldrich | P9691 |
| Anti-CD3 mAb GMP grade | OKT3 | Takara Bio | T210 |
| DNAse I | N/A | Thermo Fisher Scientific | DP (LS002139) |
| SYTOX green | N/A | Thermo Fisher Scientific | S7020 |
| RBC lysis buffer 10x | N/A | BioLegend | 420301 |
| Rabbit mAbs to CD3 | SP7 | Thermo Fisher Scientific | MA5-14524 |
| Mouse mAbs to CD8α | 1G2B10 | Proteintech | 66868-1-lg |
| Mouse mAbs to CD66b | G10F5 | Thermo Fisher Scientific | 12-0666-42 |
| Rabbit mAbs to HistoneH3 | citrulline R2+R8+R17 | Abcam | ab5103 |
| Blocking One Histo | N/A | Nacalai Tesque | 06349-64 |
| Signal enhancer HIKARI for Immunostain Solution B | N/A | Nacalai Tesque | 02375-34 |
| Histofine Simple Stain MAX-PO(M) Kit for Mouse | N/A | Nichirei | 424131 |
| Histofine Simple Stain MAX-PO(R) Kit for Rabbit | N/A | Nichirei | 424141 |
| ImmPACT®AMECRed Substrate Kit (AEC) | N/A | Vector Laboratories | SK-4285 |

**Supplementary Table 2 Univariate and multivariate analyses of the correlation between variables and disease-free survival (A) or overall-survival (B) of patients with colorectal cancer**

(A)

|  | **Univariate analysis** |  |  | **Multivariate analysis** |  |
| --- | --- | --- | --- | --- | --- |
| **Variable** | **HR (95% CI)** | **P-value** |  | **HR (95% CI)** | **P-value** |
| Pathological type | 4.567 (2.437-8.559) | <.001 |  | 1.947 (1.286-2.948) | .002 |
| Pathological T-stage | 6.144 (4.797-7.869) | <.001 |  | 4.186 (2.880-6.086) | <.001 |
| Pathological N-stage | 3.927 (2.976-5.182) | <.001 |  | 2.854 (2.071-3.399) | <.001 |
| Neo adjuvant therapy | 2.508 (1.482-4.243) | <.001 |  | 2.238 (1.553-3.225) | <.001 |
| Adjuvant therapy | 2.615 (1.941-3.522) | <.001 |  |  |  |
| Type2 diabetes mellitus | 1.437 (1.018-2.030) | .036 |  | 1.517 (1.094-2.105) | .013 |
| Metformin use | 0.303 (0.159-0.577) | .029 |  | 0.237 (0.074-0.765) | .016 |

(B)

|  | **Univariate analysis** |  |  | **Multivariate analysis** |  |
| --- | --- | --- | --- | --- | --- |
| **Variable** | **HR (95% CI)** | **P-value** |  | **HR (95% CI)** | **P-value** |
| Age | 2.026 (1.501-2.734) | <.001 |  | 1.908 (1.380-2.638) | <.001 |
| Pathological type | 5.120 (2.403-10.91) | <.001 |  | 2.230 (1.361-3.652) | .001 |
| Pathological T-stage | 2.301 (1.708-3.009) | <.001 |  | 1.709 (1.190-2.454) | .004 |
| Pathological N-stage | 3.008 (2.173-4.164) | <.001 |  | 3.154 (2.168-4.588) | <.001 |
| Neo adjuvant therapy | 1.852 (0.981-3.496) | .057 |  | 1.725 (1.017-2.927) | <.043 |
| Adjuvant therapy | 1.349 (0.953-1.910) | .091 |  | 0.583 (0.388-0.877) | .010 |
| Type2 diabetes mellitus | 1.579 (1.020-2.444) | .041 |  | 1.334 (0.894-1.992) | .158 |
| Metformin use | 0.884 (0.384-2.062) | .785 |  | 0.237 (0.074-0.765) | .016 |

Abbreviations: CI, confidence interval; HR, hazard ratio.

P values were calculated with Cox regression analysis.

**Supplementary Figure 1**

**
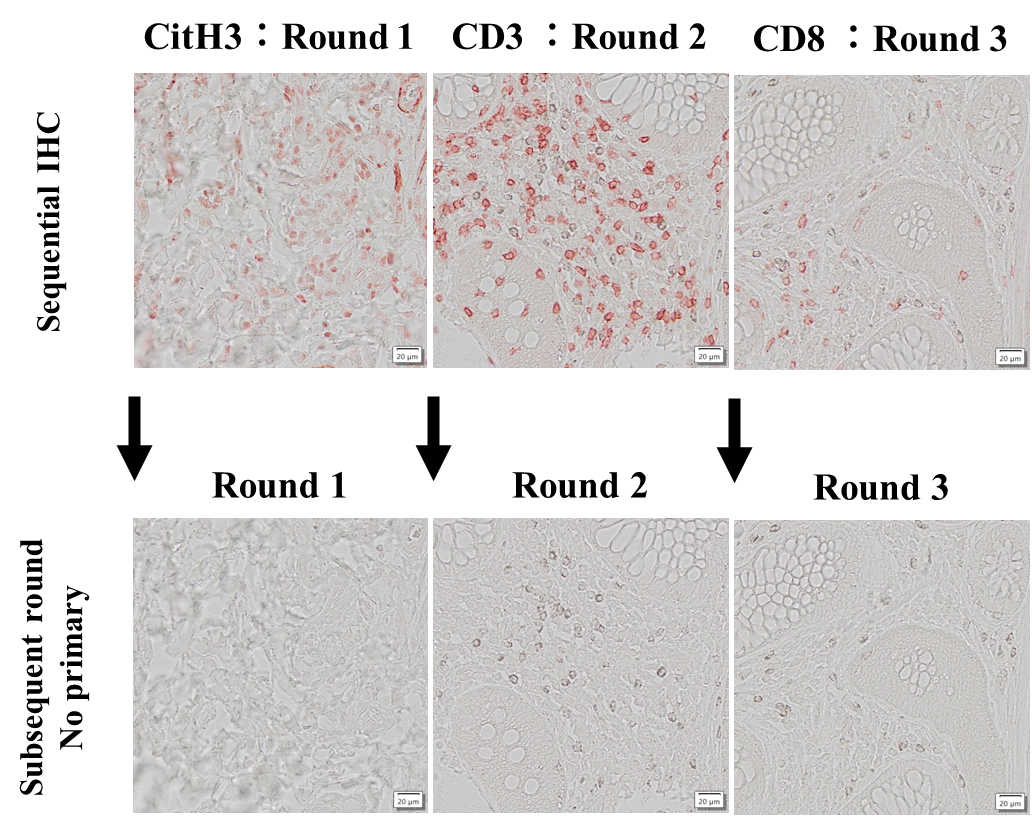
**

To validate antibody-stripping, staining was performed in the same number of rounds as described in Materials and Methods. Images represent chromogenic staining with labeled biomarkers and the number of round (top panel). Following wash of 3-Amino-9-ethylcarbazole (AEC) and antibody stripping, complete removal of antibody was confirmed by incubating with only the detection reagent and AEC in the next sequential round (bottom panel)

**Supplementary Figure 2.**


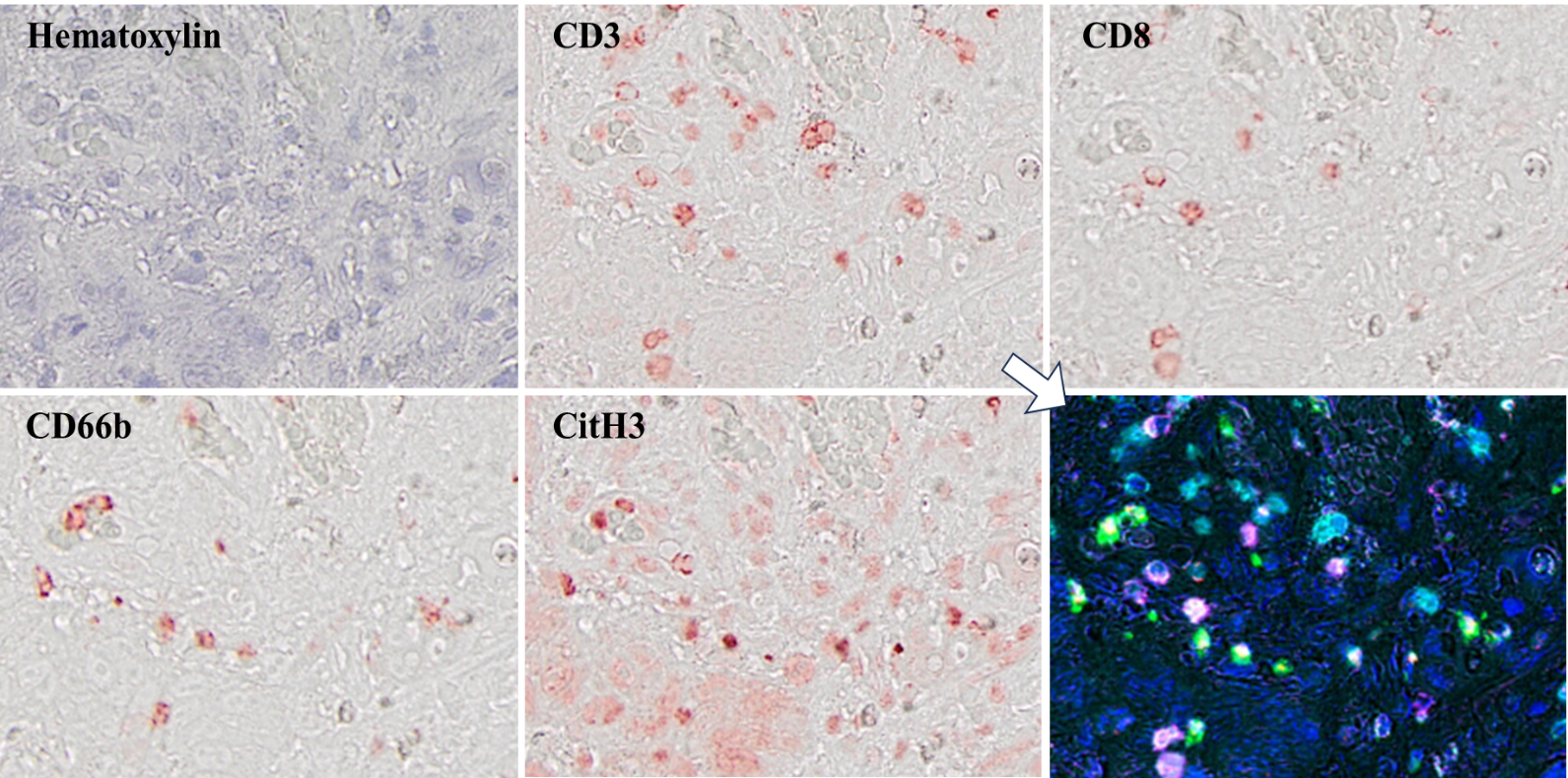


After chromogenic sequential immunohistochemical staining, images for Hematoxylin, CD3, CD8, CD66b, Cit-H3 were converted to digitized pseudo-colored images (Hematoxylin:Blue, CD3:Cyan, CD8:Magenta, CD66b:Green, Cit-H3:Red). Those images were sequentially co-registered to ensure precise alignment of cell features at the single-pixel level, utilizing the CellProfiler version 2.1.1 pipeline 'Alignment_Batch.cppipe,' and then merged using ImageJ Fiji (Right Lower Panel).
